# First person – Nichole Link

**DOI:** 10.1242/dmm.050723

**Published:** 2024-02-28

## Abstract

First Person is a series of interviews with the first authors of a selection of papers published in Disease Models & Mechanisms, helping researchers promote themselves alongside their papers. Nichole Link is first author on ‘ A Zika virus protein expression screen in *Drosophila* to investigate targeted host pathways during development’, published in DMM. Nichole conducted the research described in this article while a postdoctoral researcher in Hugo Bellen's lab at Baylor College of Medicine, Houston, USA. She is now an assistant professor and has her own laboratory at the University of Utah in Salt Lake City, investigating genetic and developmental models of human disease.


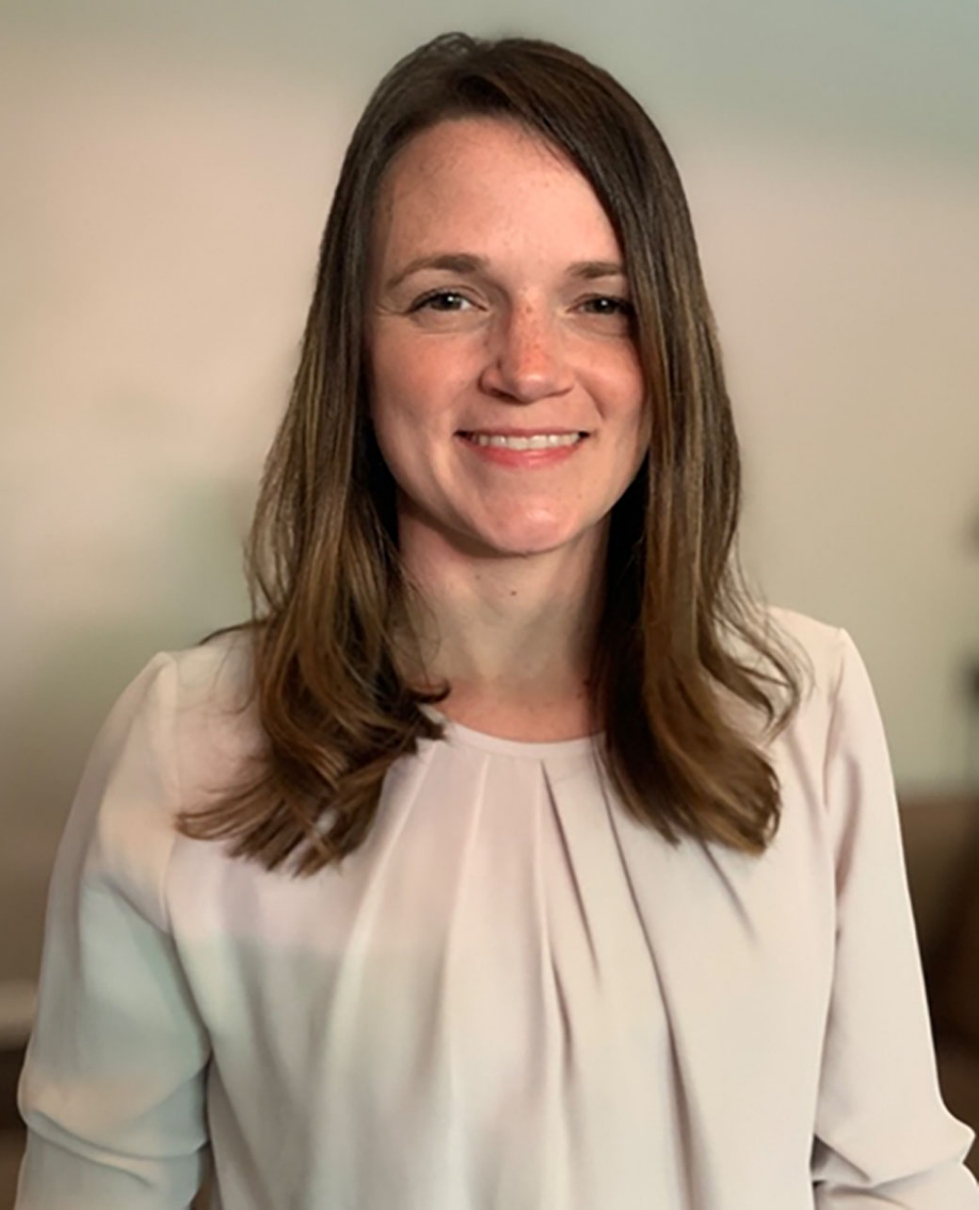


**Nichole Link**

**How would you explain the main findings of your paper to non-scientific family and friends?**

We are interested in diseases that cause problems with brain development. Genetic mutations and viral infections are often causes of these diseases. Zika virus was recently identified as a virus that can cause neurodevelopmental defects, but it was not clear how virus infection disrupts the brain. We used the fruit fly as a developing ‘test tube’ to determine how viral proteins affect host cells. When virus proteins are present, they interact with host proteins and sometimes stop their normal function, leading to unique characteristics we can see in the animal. This work looked at all Zika virus proteins and how they disrupt tissues in the developing animal. Our results show that Zika virus has the potential to inhibit conserved pathways that regulate brain development, peripheral nervous system development, eye development and neuronal function.

**What are the potential implications of these results for your field of research?**

Zika virus has been linked to small brain phenotypes (microcephaly) and other neurological disease, so it is very important to determine how this virus inhibits critical pathways during development. No treatment is currently available for Zika virus infection or the disease phenotypes that can result from infection. Although there is not an active outbreak at present, there is always the possibility that one can occur in the future. If we can determine which pathways are affected by Zika virus infection, we might be able to block how the virus interacts with those pathways to prevent disease. There are other viruses very similar to Zika virus, so the lessons we learned from this study could be applicable to related viruses or inherited genetic disorders that cause brain size defects.

**What are the main advantages and drawbacks of the experimental system you have used as it relates to the disease you are investigating?**

The fruit fly model is fast, easy to manipulate and cheap to work with. Many of our proteins work the same in the fly, so lessons we learn by using the fly can often be applied in other animals. As there are so many useful tools readily available, we can quickly dig into mechanisms of how a protein or pathway works. These details are critical for therapeutic development. Although many of the cellular pathways are conserved between humans and flies, the morphology of the brain is different. As a result, there are different cell types in the brain of flies and humans and they are organized differently. The fly isn't a natural host for Zika virus, so infection studies may not be directly applicable to humans. Our study uses protein overexpression in a model in which we assume infection has already occurred, so we skip the infection step and go straight to the cell biology.

**What has surprised you the most while conducting your research?**

Two important things! Overexpression of exogenous proteins can often cause non-specific, toxic phenotypes. We were worried about this issue because we are expressing viral proteins. We were excited to see that our results show each Zika virus protein has a very unique set of phenotypes in different tissue types. Moreover, some Zika virus proteins don't have any phenotype at all in any tissue that we analyzed, so these proteins can serve as a great control. Second, we previously found that a Zika virus protein that causes a small brain phenotype interacts with a protein, ANKLE2, that was linked to a severe brain developmental disease (microcephaly). Using the fly, we showed that the Zika virus protein inhibits the function of ANKLE2 and we could rescue brain size by overexpressing ANKLE2. This paper replicated these findings with another strain of Zika virus, validating our previous findings. These results show how useful the fly can be to identify important proteins linked to both genetic and infectious disease.

**Figure:**
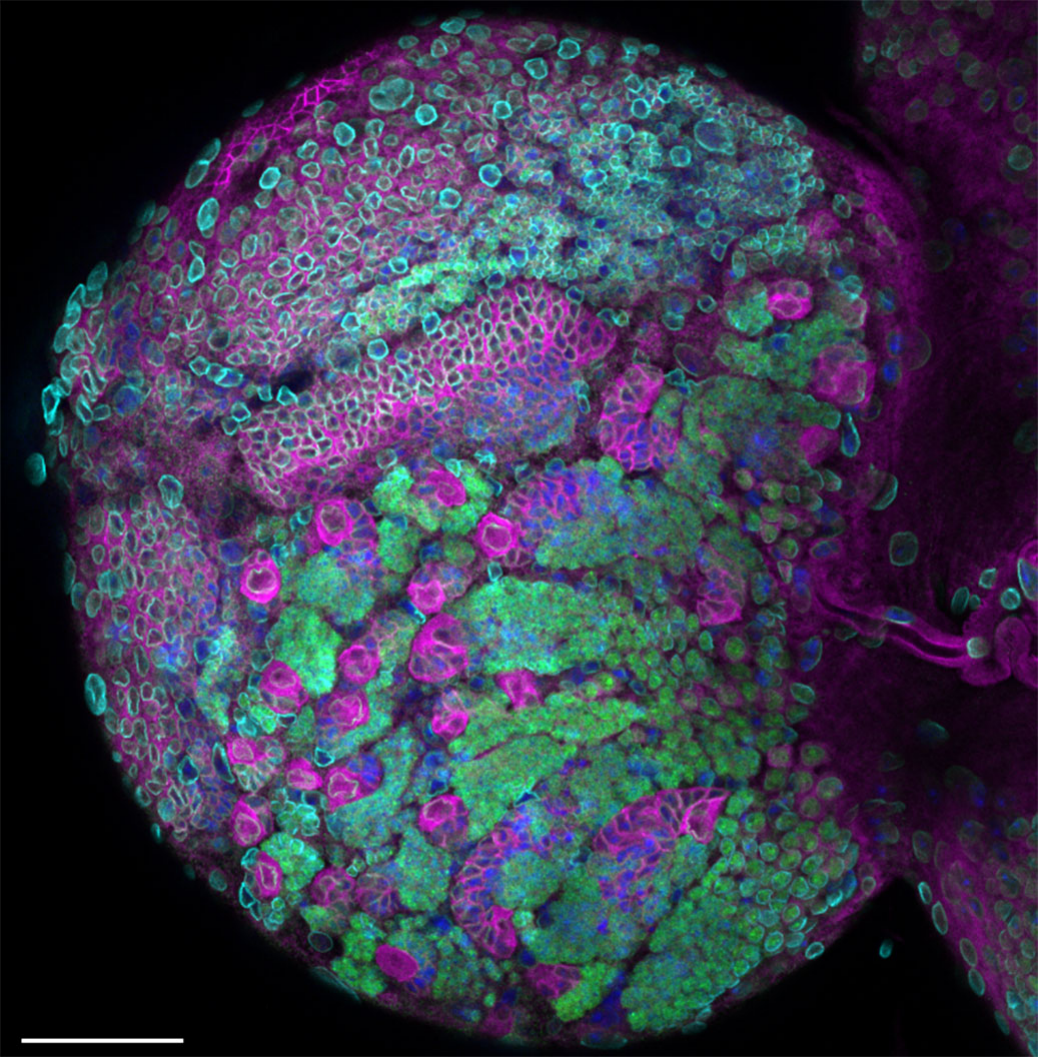
***Drosophila* third instar larval brain stained with aPKC (magenta), Elav (green, neurons), lamin (cyan, nuclear envelope) and DAPI (blue, DNA).** The large magenta cells are neural stem cells, whereas the smaller green cells are differentiating neuronal lineages produced by an asymmetrically dividing stem cell. The image shows a partial *z*-projection. Scale bar: 50 µm.

**What do you think is the most significant challenge impacting your research at this time and how will this be addressed over the next 10 years?**

We now have great resources that tell us which proteins Zika virus interacts with in our cells and what phenotypes Zika virus proteins cause when expressed. The biggest next challenge will be merging these two data sets to identify relevant interactions that cause disease. Luckily, our model system is ideal for screening. We are currently testing several top candidates identified from protein-protein interaction studies to identify which pathways Zika virus proteins inhibit to cause disease. Once we identify targets, we will have to determine how to block virus-host interactions in a way that can be used in humans.

**What is one thing you would highlight that was important for the success of your research?**

Collaboration. This work was a great collaborative effort between our lab, the Yamamoto lab and essential foundational work from the Shah lab. This work could not have been done without our groups coming together, sharing data and helping each other. Much of the work we do is based on great collaborations, and our work is truly enhanced by so many different experts contributing to the same end goal.

It seems that more mentor-mentee relationships are moving to a caring and supportive nature […] Finding a group of mentors that support you and promote you can be critical in all career stages.

**What changes do you think could improve the professional lives of scientists?**

Increased support and compensation. It seems that more mentor-mentee relationships are moving to a caring and supportive nature rather than one of ‘tough love’ or ‘eat or be eaten’ structure. However, you can still encounter this attitude, and it can break the confidence of trainees. Finding a group of mentors that support you and promote you can be critical in all career stages. Compensation has been a hot topic for all levels of scientists in academia. The better we can pay our trainees, the less chance of losing talented scientists.

**What's next for you?**

I started an independent position at the Department of Neurobiology at the University of Utah as an assistant professor in the fall of 2020. It was a really tough time to establish a lab, but we persevered. I am thankful that we have a great group that works really hard. Everyone is doing stellar work and making serious progress. I am so excited to see how our lab grows and publishes in the next few years. We have projects in the works on neural stem cells, aging, previously unreported causes of human disease, and how viruses hijack host pathways to cause disease. So, the next step for me is to provide the necessary mentorship to trainees in the lab so that they have the tools to be successful.
